# Satellite myoblast and mesenchymal stem cell injections decrease fatty degeneration after rotator cuff tear in rats

**DOI:** 10.1002/jeo2.12087

**Published:** 2024-07-24

**Authors:** Tahir Koray Yozgatli, Elif Gelenli Dolanbay, Tunca Cingoz, Ahmet Emre Paksoy, Unal Uslu, Ercument Ovali, Baris Kocaoglu

**Affiliations:** ^1^ Department of Orthopedic Surgery Acibadem University Faculty of Medicine Istanbul Turkey; ^2^ Department of Histology and Embryology Istanbul Medeniyet University Faculty of Medicine Istanbul Turkey; ^3^ Department of Orthopedic Surgery Atatürk University Faculty of Medicine Erzurum Turkey; ^4^ Acibadem Labcell Cellular Therapy Laboratory Istanbul Turkey

**Keywords:** fatty degeneration, rotator cuff, stem cell therapy, surgical repair

## Abstract

**Purpose:**

Rotator cuff (RC) tears cause fatty degeneration, aggravated by delayed treatment. Surgical repair alone cannot reverse fatty degeneration. It was aimed to test if local injections of satellite cell‐derived myoblasts or satellite myoblasts (SM) from the deltoid region and mesenchymal stem cells (MSCs) from the subcutaneous abdominal fat pad would stimulate myogenesis and decrease adipogenesis in the rat model of fatty degenerated RC tear.

**Methods:**

A standardized RC tear surgery was performed on both shoulders of 24 Wistar albino rats at *t* = 0, and rats were followed for 8 weeks to create a chronic degeneration model. The animals were randomly divided into repair + SM and MSC (*n* = 12) or repair only (*n* = 12) groups. Transosseous repair with or without stem cell‐based injection was performed on the right shoulder of all rats on week 8, with additional injections on weeks 9 and 10. The left shoulders were used as control. The animals were followed until week 14 for recovery.

**Results:**

Histological and histomorphometric analyses were performed in week 14. The repair + SM and MSC group had a significantly greater supraspinatus muscle mass than the repair only and control groups. The adipose tissue ratio was significantly lower in the repair + SM and MSC groups versus the repair only and control groups.

**Conclusion:**

Histologically, the repair + SM and MSC group had improved muscle and tendon organization. In treating chronically degenerated RC tear in a rat model, surgical repair combined with injections of SM and MSC improved fatty degeneration, tendon healing and myogenesis.

**Level of Evidence:**

Level III.

## Graphical abstract

Satellite myoblast (SM) and mesenchymal stem cell (MSC) injections improve fatty degeneration reversal after rotator cuff tear in rats. The experimental design shows the details of interventions performed on the control, repair only and repair + SM and MSC groups. The plots in the results section show a decrease in the adipose tissue ratio and an increase in the muscle tissue ratio in the repair + SM and MSC group. The asterisks signify a statistically significant difference. The histological preparations show the differences in the adipose tissue and muscle tissue ratios between the groups.

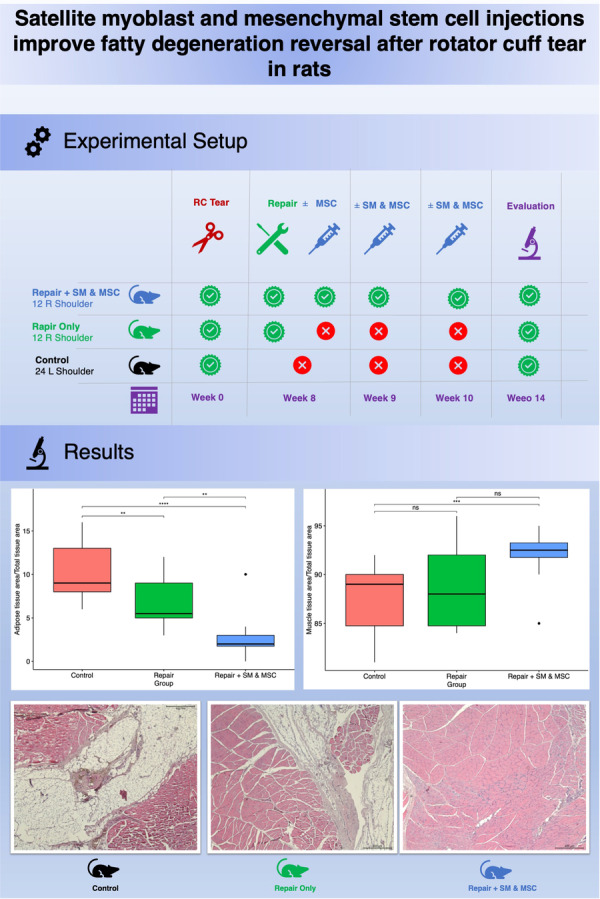

**Abbreviations**
ADSC: adipocyte‐derived stem cell
DMEM: Dulbecco's modified Eagle's medium
FBS: foetal bovine serum
H&E: haematoxylin–eosin
IM: intramuscular
MSC: mesenchymal stem cells
PBS/wo: phosphate‐buffered saline solution without calcium or magnesium
PSCs: perivascular stem cells
RC: rotator cuff
SM: satellite myoblasts
SPSS: Statistical Package for Social Sciences
TCM: trichrome Masson stain

## INTRODUCTION

Rotator cuff (RC) tears can cause significant pain and functional impairment due to the transformation of the muscle microarchitecture [8, 9, 37]. If an RC tendon tear is left untreated, the muscle will undergo fatty degeneration. Early surgical repair can prevent fatty degeneration from progressing, but delayed repair cannot decrease fatty degeneration that has already advanced [13, 30, 35]. Advanced fatty degeneration is considered a contraindication to surgical repair because it correlates with poor postrepair results [8, 9, 37]. Up to 94% of patients with Goutallier grade 3 or 4 fatty degeneration experience failure to heal, retear or loss of function after surgical repair [8, 9, 37]. Stem cell‐based injections are a promising new therapeutic solution for regenerating muscle tissue in advanced fatty degeneration [27].

Different stem cell types have unique plasticity profiles and functions. For example, mesenchymal stem cells (MSCs) can differentiate into myocytes, adipocytes and chondrocytes, whereas satellite cell‐derived myoblasts or satellite myoblasts (SMs) can differentiate into skeletal myocytes and support the muscle fibres [20, 33]. MSC‐based therapies can improve muscle function after tendon repair and help decrease fatty infiltration within the muscle belly [14]. A previous study showed that the combination of SMs and MSCs had a successful outcome in numerous myocardial ischaemia models [28]. Local application of MSCs and cultivated myocytes improve the regeneration of damaged cardiomyocytes by increased angiogenesis with vascular endothelial growth factor, increased myogenesis with myocardial‐like cells and improve pump function [3].

Previously, Güleçyüz et al. showed that local injection of MSCs into the fatty degenerated supraspinatus muscle increased its mass However, they did not confirm the improvement in the muscle histologically [14]. Furthermore, they harvested myocytes from the gastrocnemius muscle of the animals. Thus, two separate anatomic locations were dissected for MSC harvesting and tendon repair. Considering the additional burden of separate incisions, simultaneous stem cell harvesting and tendon repair could be more convenient and practical. SMs can be conveniently obtained from the deltoid muscle neighboring the RC, while MSCs can be extracted from the subcutaneously accessible abdominal fat pad, applicable in both rat models and humans [1, 2, 4, 25, 29]. To our knowledge, no previous studies have explored the utilization of the shoulder region for stem cell harvesting in the rat RC fatty degeneration model. To investigate the change in fatty degeneration, we established a novel stem cell culture model utilizing SMs from the deltoid muscle and MSCs from the abdominal fat pad. These cultured stem cells were subsequently injected into fatty degenerated RCs, allowing us to evaluate their efficacy in slowing the degenerative process.

The aim of this study was to test if delayed repair of a tendon tear and SM and MSC injection into the fatty degenerated RC could halt muscle degeneration by stimulating myogenesis, decreasing adipogenesis and improving tendon healing in a rat model. To determine the effect of the intervention on myogenesis and adipogenesis, the muscle compositions of SM‐ and MSC‐injected animals and controls were grossly and microscopically compared. The hypothesis was that local stem cell injection would increase the gross muscle mass, decrease the amount of fatty tissue and promote tendon‐to‐bone healing in the fatty degenerated RC tear model.

## MATERIALS AND METHODS

### Animals

The rat model was chosen for this study because the shoulder anatomy of a rat is similar to that of a human [17, 23]. Ethical approval was obtained for the use of laboratory animals from the University Research Ethics Commission for Animal Experiments. The experiment included 24 male Wistar albino rats (equivalent to 48 shoulders), while two additional rats were specifically utilized for allogeneic stem cell isolation and culture. The animals were housed in a pathogen‐free facility with optimal temperature (25°C), lighting (12 L:12 D) and ad libitum access to food and water.

### Study design

The animals were randomly assigned to the repair + SM and MSC (Group 1) and repair only (Group 2) groups. The body masses of the animals in each group were between 0.3 and 0.4 kg at the time of randomization. A bilateral RC tear model was established in all animals. The right shoulders of all animals were used for the treatment, while the left shoulders served as the control group (no treatment). Two additional rats were euthanized, and SM and MSC isolation was performed as follows: SM isolation: the deltoid muscles were isolated and biopsied, and SMs were cultivated from the biopsy samples. In MSC isolation, the abdominal fat pads were isolated and biopsied, and MSCs were cultivated from the biopsy samples. These two rats were not included in the experiment groups.

**Figure 1:**
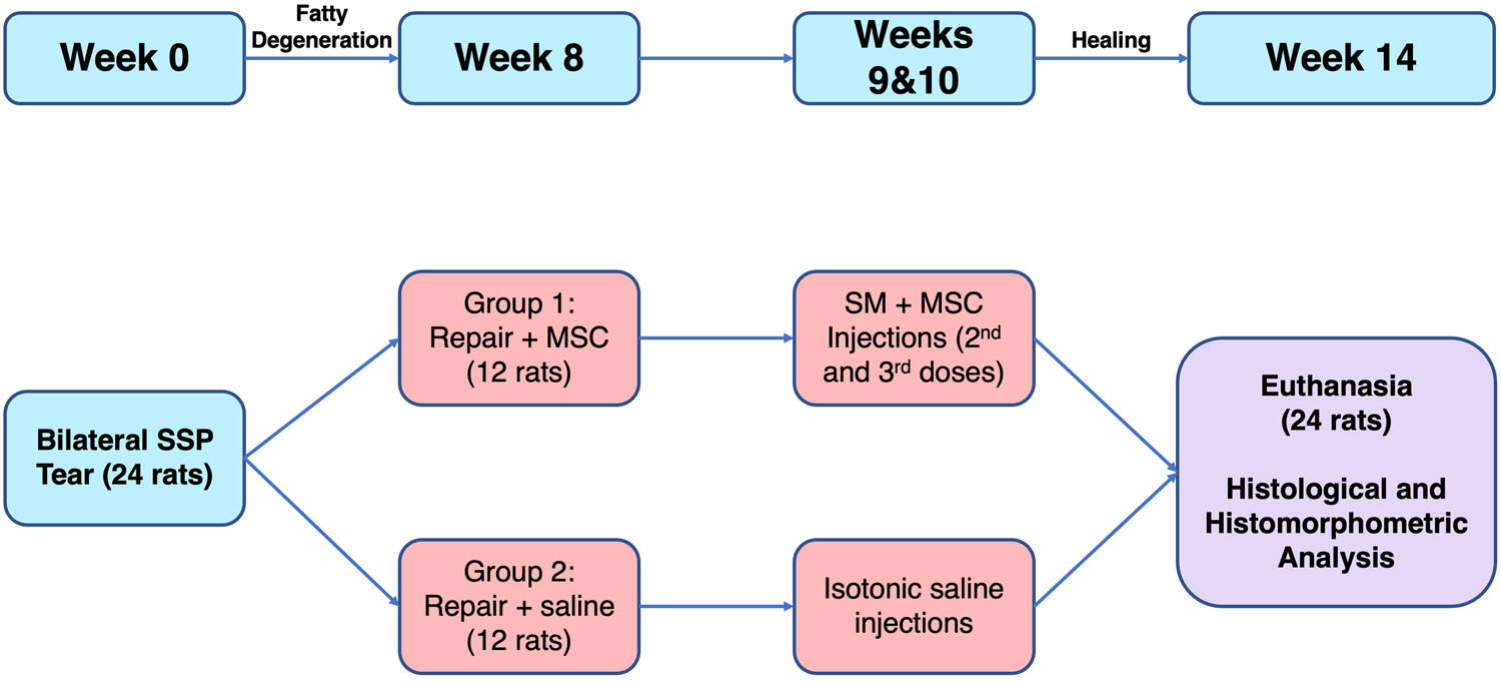
Flowchart of the experimental setup. MSC, mesenchymal stem cell; SM, satellite myoblast; SSP, supraspinatus.

The tendon defect surgery, tendon repair, stem cell treatment and histological evaluation phases of the study were performed at specific times over 14 weeks. (Figure 1). At time zero, all rats in the experiment groups (*n* = 24) underwent a standardized tendon defect surgery to create a full‐thickness tear in the supraspinatus tendons. The animals were observed for eight weeks after the procedure to obtain adequate fatty degeneration before performing the surgical repair [11, 20]. Group 1 (repair + SM and MSC, *n* = 12) underwent repair with an injection of MSCs into the supraspinatus muscle. The injection was performed under direct visualization into the distal 1/3rd of the muscle belly. Group 2 (repair only, *n* = 12) underwent repair with an injection of saline solution into the supraspinatus muscle at the same location. In the postoperative weeks 1 and 2, the animals in Group 1 were injected with MSCs, this time in combination with SMs. The animals in Group 2 were injected with saline solution. These last two injections were performed into the muscle by palpating the proximal humerus and performing the injection through the surgical site aiming for the muscle belly of the supraspinatus. Thus, a total of three stem‐cell or saline injections were performed. All rats were euthanized four weeks after the last injection (Postoperative Week 6). All animals' supraspinatus muscle and tendon muscle junctions were isolated, and their weights were measured. The tissue samples were stored at −80°C until the histological analysis. The microscopic fat, muscle and tendon tissue levels of the two treatment groups and the control group were compared.

### Tendon defect surgery

At time zero, standardized surgery was performed for full‐thickness supraspinatus tendon tears on all animals (24 animals, 48 shoulders). Anesthesia was induced using an intraperitoneal injection of sodium thiopental (50 mg/kg Pentothal; Abbott Laboratories). After induction of anesthesia, a 2 cm incision was made craniocaudally over the shoulder, exposing the deltoid muscle. The deltoid muscle was split, and the supraspinatus tendon was identified. A full‐thickness tendon tear was made at its insertion site with a number 15 blade. The distal part of the muscle was braided and fixed to the trapezius muscle using a 5/0 monofilament polypropylene suture (Prolene; Ethicon) to prevent spontaneous healing and provoke muscle atrophy and induce fatty degeneration of the muscle. Additionally, the tendon attachment site of greater tuberosity on the humerus was covered by placing tubercular resin to prevent spontaneous healing. A postoperative dose of intramuscular (IM) cefazolin sodium (0.1 mg/kg Cefazolin; Pfizer) was injected for infection prevention. All rats received IM buprenorphine injections (0.030 mg/kg Buprenex Bedford Laboratories) for postoperative analgesia and were followed in single cages with ad libitum access to food and water.

### Tendon repair surgery and stem cell treatment

Each animal was weighed prior to the tendon repair operation. The animal was prepared as described in the tendon defect surgery section. The polypropylene suture that was used to fixate the supraspinatus tendon to the trapezius was removed. The scar tissue developed in the anatomical supraspinatus attachment site on the humerus was removed. Two 0.5 mm tunnels were drilled through the greater tuberosity. A 5/0 monofilament polypropylene (Prolene; Ethicon) suture was passed from the supraspinatus and the tunnels. The distal ends of the suture were tied, completing standard transosseous tendon repair. At this point in the surgery, Group 1 was injected with a 200‐μL injection of MSCs with 3 × 10^6^ (±%10) cells/mL suspended in (first injection: adipose tissue‐derived MSCs), and Group 2 received an equal amount of saline injection. The injections were performed into the distal 1/3rd of the muscle belly under direct vision. Postoperative antibiotic prophylaxis and analgesia were administered as previously described.

### Stem cell isolation and culture

#### Adipose tissue‐derived MSC isolation and culture

The two rats not included in the experiment groups were used for stem cell isolation and culture. The two animals were prepared as previously described. Anaesthesia was induced, and an intracardiac injection of 1 mL (16 g/100 mL) pentobarbital Narcoren (Merial GmbH) was performed for euthanasia. The subcutaneous adipose tissues were harvested from the abdominal region of the rats and placed in a phosphate‐buffered saline solution without calcium or magnesium (PBS/wo) containing 1% penicillin/streptomycin. The tissues were taken into a sterile biosafety cabinet and washed. Later, the tissues were transferred into a petri dish, 10 cm in diameter, and very finely chopped into 1 mm × 1 mm cubes using sterile forceps and scissors. The minced adipose tissues were placed into a solution containing collagenase type 1 at a concentration of 2.5 mg/mL and incubated for 90 min at 100 rpm in a 37°C mixer. After incubation, the enzymatically degraded tissues were passed through a 70 µm strainer and centrifuged at 250*g* for 10 min.

After discarding the supernatant, the cell pellet was suspended with PBS/wo and centrifuged at 250*g* for 10 min. The process was repeated for a total of three washes. The supernatant was discarded, and the pellet was suspended with Dulbecco's modified Eagle's medium (DMEM) F12 containing 10% foetal bovine serum (FBS). At this stage, the sample was taken for sterility and cell number count and cultivated as 1 × 10^6^ cells/T‐75 flask (10 mL). The samples were incubated at 37°C and 5% CO_2_ for 24 h, and the first medium change was performed. Thereafter, the medium was changed every 2 days. The cell proliferation was followed with an inverted microscope. The cells were passaged with trypsin/EDTA when they covered 70% of the flask base. For the injections, the cells obtained from passage 2 were used. The injections were performed with the cells in the growth medium.

#### MSC characterization, sterility checks and cell count

Following passage 2, the cells were subjected to incubation with 1 μg of antibodies conjugated with R‐phycoerythrin, fluorescein isothiocyanate and allophycocyanin or isotype‐matched control immunoglobulin Gs at room temperature for 45 min. Subsequently, the samples underwent analysis using a FACSCalibur flow cytometer (BD Biosciences) after the completion of incubation and washing steps. The antibodies used for MSC identification included CD45, HLA‐DR, CD34, CD90, CD105 and CD73. The sterility checks were performed twice. The first check was performed using the minced tissue sample before the culture process. The second check was performed using the culture sample three days prior to the injections. The samples from the minced tissue and culture sample were incubated in BACTEC™ (BD Diagnostics) aerobe and anaerobe culture media for microbiology cultures. The cultures were followed for 3 days to check for bacterial growth signals. If there was no bacterial growth signal for 3 days in the minced tissue, the stem cell culturing process was continued. Similarly, if there was no signal at 3 days in the culture medium, the cells were used in the experiment. The microbiological cultures were followed for a total of 2 weeks and the results were confirmed. For the cell count process, a volume of 100 μL of cell suspension was mixed with an equal amount of a working solution of trypan blue and then transferred to a hemacytometer chamber. Cell counting was performed using a microscope with a magnification of 10 × 40.

#### Deltoid region‐derived SM isolation and culture

The animals were prepared as previously described. The skin overlying the deltoid region was removed with a No. 15 scalpel. The deltoid muscle was removed and treated with 1% penicillin/streptomycin. The tissues were placed in PBS/wo solution in a Petri dish, and the Petri dish was transferred to a sterile biosafety cabinet. The muscle tissue was minced and prepared as previously described for the fat tissue. The minced muscle tissues were taken into 2.5 mg/mL collagenase and 0.3 mg/mL hyaluronidase containing DMEM solution at 37°C and incubated at 100 rpm for 90 min on a mixer. The enzymatically disintegrated tissues were passed through a 70 µm filter and centrifuged at 300*g* for 5 min. The supernatant was discarded, and the cell pellet was suspended in PBS/wo and centrifuged for 5 min at 300*g*. The process was repeated for a total of three washes. The resulting pellet was suspended in DMEM containing 10% FBS. The sterility checks and cell count were performed at this stage as previously described, and the samples were placed in flasks at 1 × 10^6^ cells/T‐75 flask (10 mL). The samples were incubated at 37°C and 5% CO_2_ for 24 h, and the first medium change was performed. Thereafter the medium was changed every 2–3 days. The cell proliferation was followed with an inverted microscope. The cells were passaged with trypsin/EDTA when they covered 70% of the flask base. For the injections, the cells obtained from passage 2 were used. The injections were performed with the cells in the growth medium.

The rationale for the specifics of the injection was as follows: the first injection was performed with only MSCs (3 × 10^6^ [±%10] cells/mL) and the subsequent two injections with SMs (3 × 10^6^ [±%10] cells/mL) and MSCs (3 × 10^6^ [±%10] cells/mL). The study was designed to utilize the MSCs in all three injections because the abdominal fat pad‐derived MSCs can easily be cultivated and prepared prior to arthroscopic shoulder surgery. However, in the hypothetical case of a human clinical set‐up, the deltoid muscle tissue (SM source) may only be harvested without causing additional surgical burden during the arthroscopic repair. Therefore, the cultivated SMs can only be available for culture at the time of the surgery and can only be injected at the second and third postoperative injections.

#### Removal of the supraspinatus muscle and tendon

Each animal was weighed prior to the muscle and tendon unit removal. The animals were weighed using a bench scale (Kern FKB 30K1; KERN & SOHN GmbH). The anaesthesia was induced, and an intracardiac injection of 1 mL (16 g/100 mL) pentobarbital Narcoren (Merial GmbH) was performed for euthanasia. Both supraspinatus muscles and tendons were removed from the supraspinatus fossa and weighed. The tissues were weighed using a precision scale (Shimadzu AUW220D Semi‐Micro Analytical Balance; Shimadzu Scientific Instruments).

#### Histological evaluation technique

Two histologists who were blinded to the intervention performed the histological analyses. A Leica DM 4000 microscope equipped with Stereo Investigator software (Micro Bright Field Inc.) was used for histomorphometric analysis. In addition, a semiautomated stereology workstation composed of a CCD digital camera (Optronics Microfire 1600x1200P), an image capture card ATI FireGL (Advance Micro Device), a personal computer and a computer‐controlled motorized specimen stage (Bioprecision), a microactuator (Heidenhein) and a light microscope (Leica DM 4000B) were used for image and stereological analyses.

For histological examination, fresh tendons were fixed with 10% neutral buffered formaldehyde, kept at room temperature for 24 h and embedded in paraffin. After sectioning, staining was done with haematoxylin–eosin (H&E) and trichrome Masson stain (TCM) for histomorphometric analysis.

H&E staining was done. Sections (5 µm) were stained with Harris hematoxylin (Bio Optica BO 05‐06004/L) for 5 min, colour changed in tap water 5 min, 1% eosin Y (Bio Optica BO 05‐10003/L) for 7 min, running tap water, 5 min, dehydrated, cleared in xylene and mounted.

TCM staining was done (BO 04‐010802 Masson Trichrome with aniline blue). Four different stains are used: Weigert's iron hematoxylin for nuclei, picric acid for erythrocytes, a mixture of acid dyes (acid fuchsin‐‘ponceau de xylidine’) for cytoplasm and aniline blue for connective tissue. The section was brought to distilled water. Six drops of reagent A were added to the section, and six drops of reagent B were added and left to act for 10 min. The slide was washed, and 10 drops of reagent C were added to the section: for 4 min. Washed quickly (3–4 s) in distilled water and added 10 drops of reagent D to the section: Left for 4 min. Washed in distilled water and instilled 10 drops of reagent E: Left for 10 min. Drained the slide without washing and put 10 drops of reagent F into the chamber: Allowed to act for 5 min. Washed with distilled water and rapidly dehydrated with ascending alcohols, rinsing for 1 min in final absolute ethanol. Cleared in xylene and mounted.

The Quick Measure Line of this system was used to determine the areas of the tendon, striated muscle and adipose tissue. Following the paraffin embedding of the tissues, the supraspinatus tissues were divided into five regions: proximal/middle/distal muscle belly zone, proximal muscle‐tendon junction zone and distal muscle‐tendon junction zone. Four separate slides from each of the five tissue zones of each supraspinatus sample were used for the measurements, and the tendon, striated muscle and adipose tissue ratios were calculated. Two separate analyses of the slides were performed and averaged to calculate the final area percentages. Thus, the average areas of the tendon, muscle and adipose tissues of the control group, repair + SM and MSC and repair only groups, were determined.

A statistical power analysis was used to calculate the sample size with a power of 80%, a 95% confidence level and a *p* value of 0.05. Based on previous literature on average, a fatty area of 8% of the total sample was anticipated, with a standard deviation of 2.5% to be observed in the control group, and a change in area of 4% would be clinically noticeable as this would mean halving the adipose change [31]. It was determined with the power analysis results that a sample size of 12 in each group would be needed [19]. The statistical analysis was performed utilizing Statistical Package for Social Sciences (SPSS) (for Windows; version 18.0) (SPSS Inc.). Box plots were generated by the ggplot2 data visualization R package, and significance levels were added to the plots by the stat_pvalue_manual function in the ggpubr R package. The Shapiro–Wilk test was used to assess whether the data were normally distributed. The analysis of variance test was applied to determine the differences in the number of tissues, and the Duncan test was used to determine the significance between groups. The post hoc 97 multiple comparison test was used to assess the significance of the differences. A *p* value less than 0.05 was considered statistically significant.

## RESULTS

### Supraspinatus tissue histomorphometric and mass measurement

**Figure 2:**
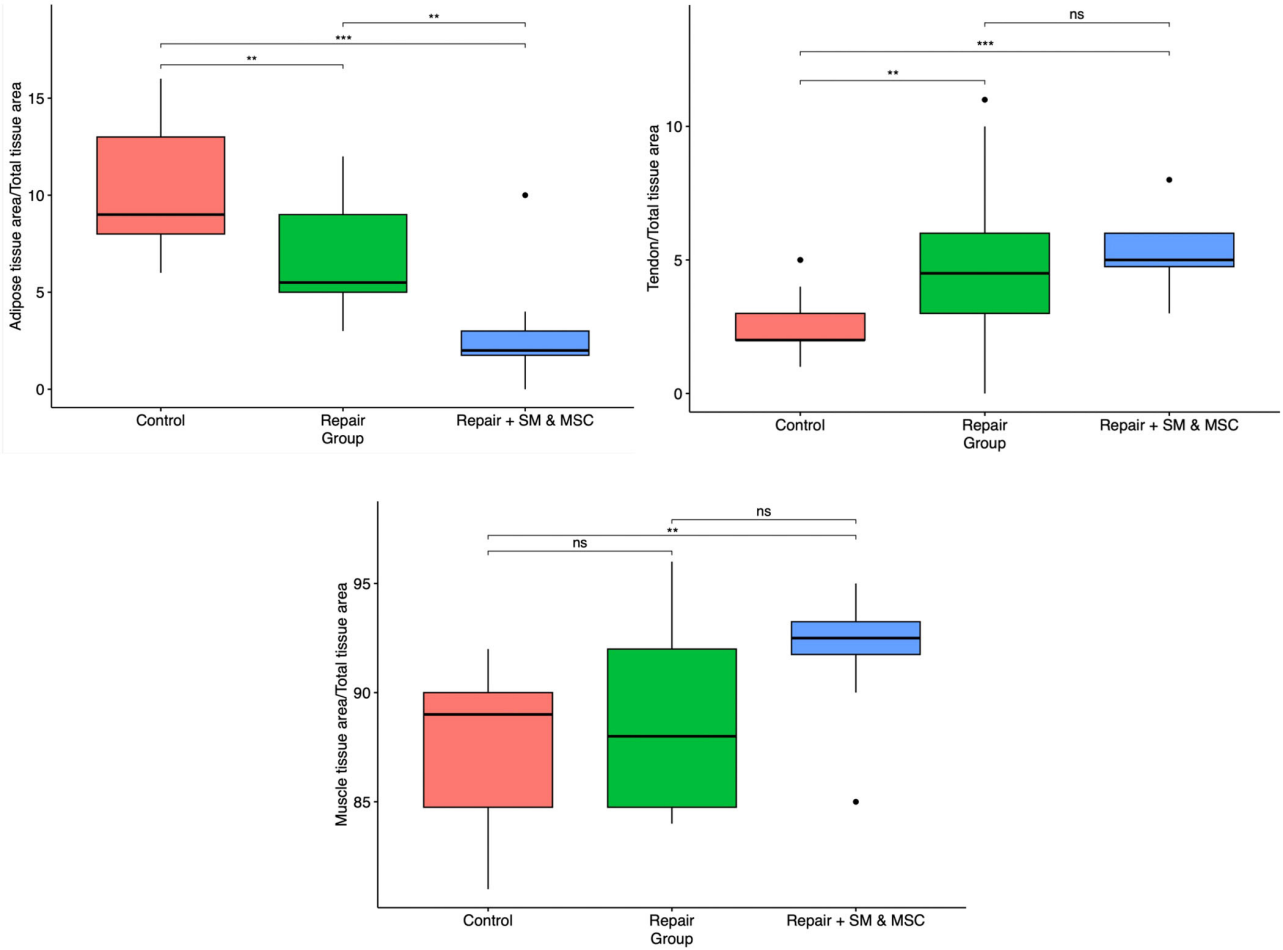
Plots of the histomorphometric analysis. From left to right, adipose tissue area/total tissue area, tendon/total tissue area, muscle tissue area/total tissue area. Control: red, repair only: green, repair + SM and MSC: blue. *p < 0.05, **p < 0.01, ***p < 0.001, ns, not significant. MSC, mesenchymal stem cell; SM, satellite myoblast.

At Week 14, the adipose, tendon and muscle tissue areas were measured, and the total tissue area ratios were calculated as previously described. Table 1 and Figure 2 depict the results of this histomorphometric analysis. The repair + SM and MSC group had a significantly lower percentage of adipose tissue compared to the control group (*p* < 0.001) and the repair only group (*p* = 0.009). The repair only group had a significantly lower ratio of adipose tissue compared to the control group (*p* = 0.004). Both the repair + SM and MSC group and repair only group had significantly greater ratios of the tendonous portion of the supraspinatus tissues compared to the control group (*p* < 0.001 and *p* = 0.002, respectively). The proportions of tendon tissues were similar between the repair + SM and MSC group and the repair only group (*p* = 0.85). The muscle ratio was significantly higher in the repair + SM and MSC group compared to the control group (*p* = 0.002).

**Table 1:** Histomorphometric analysis results of the supraspinatus muscle.

	Adipose %	*p* Values		Tendon %	*p* Values		Muscle %	*p* Values	
Group	Mean ± SD	vs. Control	vs. Repair	Mean ± SD	vs. Control	vs. Repair	Mean ± SD	vs. Control	vs. Repair
Control	10.0 ± 3.2			2.3 ± 1.2			87.7 ± 3.5		
Repair	6.5 ± 2.8	**0.004**		4.8 ± 3.2	**0.002**		88.7 ± 4.2	0.70	
Repair + SM and MSC	2.8 ± 2.5	**<0.001**	**0.009**	5.2 ± 1.3	**<0.001**	0.85	92 ± 2.6	**0.002**	0.06

*Note*: Results of the histomorphometric analysis of the supraspinatus tissues in the control (*n* = 24), repair (*n* = 12) and repair + SM and MSC (*n* = 12) groups. The bold values indicate statistically significant values.

Abbreviations: MSC, mesenchymal stem cell; *n*, number of shoulders; SD, standard deviation; SM, satellite myoblast.

The muscle mass and muscle mass as a percent of the animal weight were significantly higher in the repair + SM and MSC groups compared to the control and repair only groups.

Table 2 depicts the comparison of the masses of the supraspinatus musculotendinous units as they were weighed after dissection.

**Table 2:** Muscle mass analysis.

	Muscle mass (g)	*p* Values		Muscle/animal weight × 100 (%)	*p* Values	
Group	Mean ± SD	vs. Control	vs. Repair	Mean ± SD	vs. Control	vs. Repair
Control	5.9 ± 0.9			1.55 ± 0.18		
Repair	6.1 ± 1.2	0.81		1.63 ± 0.21	0.48	
Repair + SM and MSC	7.8 ± 1.1	**<0.001**	**<0.001**	2.00 ± 0.2	**<0.001**	**<0.001**

*Note*: Mass analysis of the supraspinatus muscle unit in the control (*n* = 24), repair (*n* = 12) and repair + SM and MSC (*n* = 12) groups.

Abbreviations: g, grams; MSC, mesenchymal stem cell; *n*, number of shoulders; SD, standard deviation; SM, satellite myoblast.

### Supraspinatus histologic evaluation

Supporting Information S1: Images 1–18 depict the histological changes in the control, repair only and repair + SM and MSC groups with H&E and TCM stains under ×5, ×20 and ×40 magnifications. Selected images of repair only and repair + SM and MSC groups are shown in Figures 3, 4, 5. Regarding tissue quality analysis, the repair only group had increased fibroblasts responsible for collagen production compared to the control group. In the repair only group, the tissue itself had mature and immature collagen bundles and inflammatory cells dispersed between myocytes, signifying a chronic inflammation state and ongoing subacute phase of wound healing. The repair + SM and MSC group had mostly mature collagen bundles and increased connective tissue within the tendon with fibroblasts and tenocytes.

**Figure 3:**
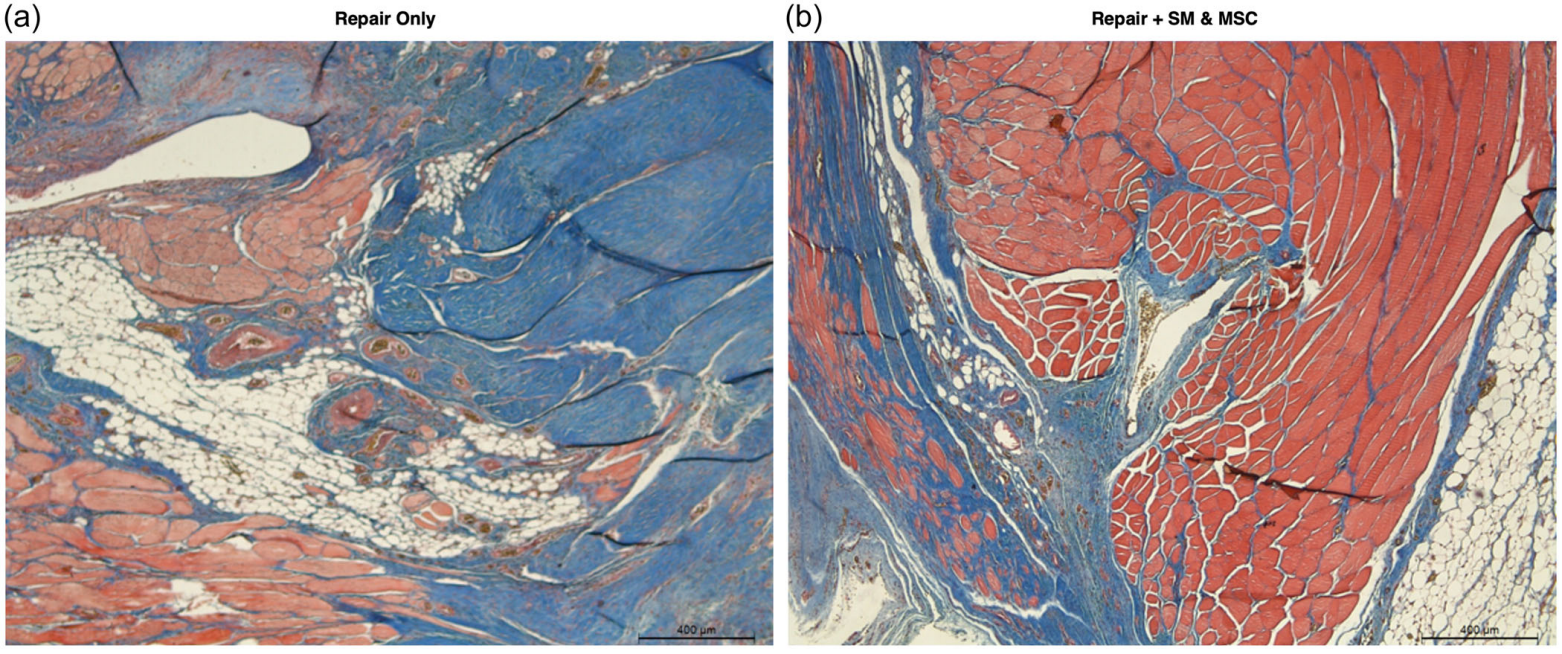
TCM stain ×5 magnification microscopic images of the supraspinatus muscles from the proximal muscle/tendon junction zone. (a) Repair only: Connective tissue integration has begun, and the collagen forming the tendon is primarily immature. (b) Repair + SM and MSC group: Well‐developed connective tissue integration, muscle tissue and tendon tissue containing mostly mature collagen fibres, markedly less adipose tissue than the repair only group. MSC, mesenchymal stem cell; SM, satellite myoblast; TCM, trichrome Masson stain.

**Figure 4:**
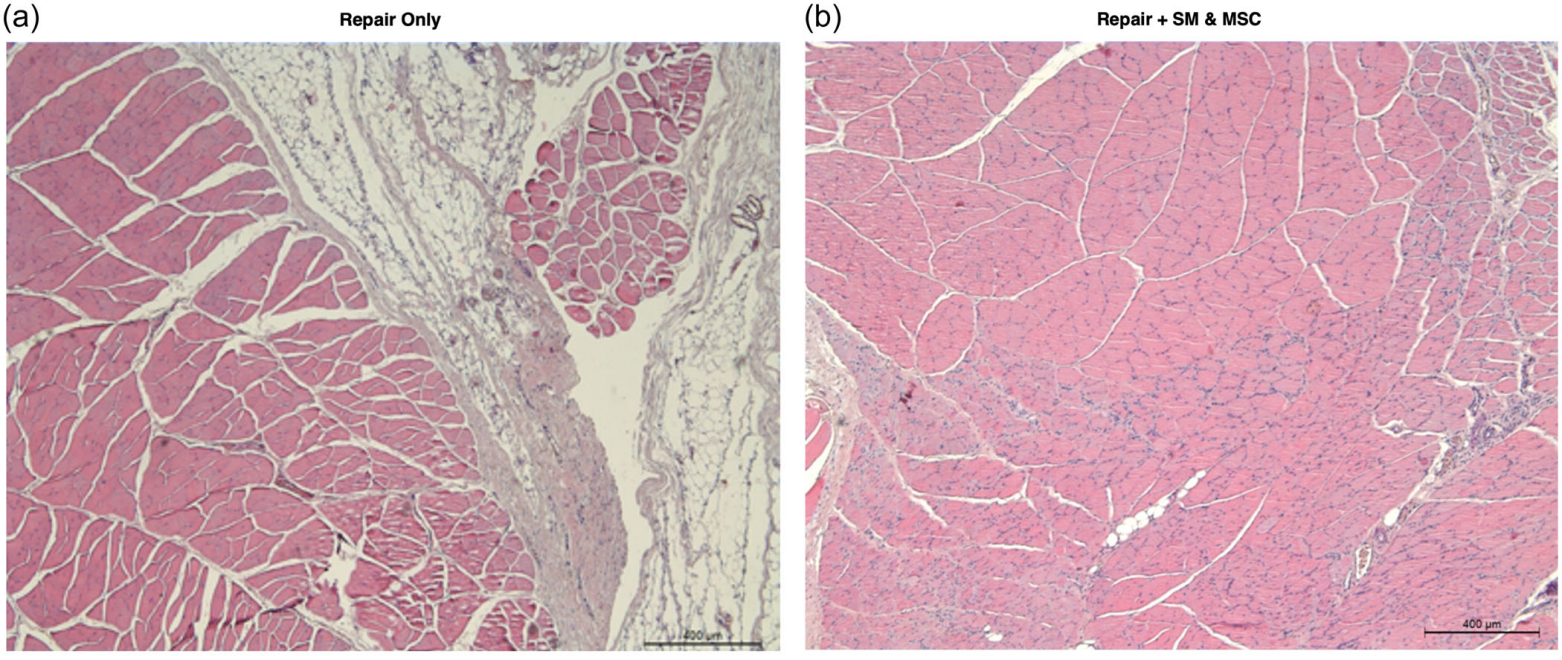
H&E stain ×5 magnification microscopic images of the supraspinatus muscles from the middle muscle belly zone. (a) Repair only group: Mononuclear cell infiltration and ongoing inflammatory processes are evident in the tendon. (b) Repair + SM and MSC group: Increased fibroblasts between the muscle fibres, markedly less adipose tissue within the muscle belly, and proper muscle tissue organization compared to the repair only group. H&E, haematoxylin–eosin; MSC, mesenchymal stem cell; SM, satellite myoblast.

**Figure 5:**
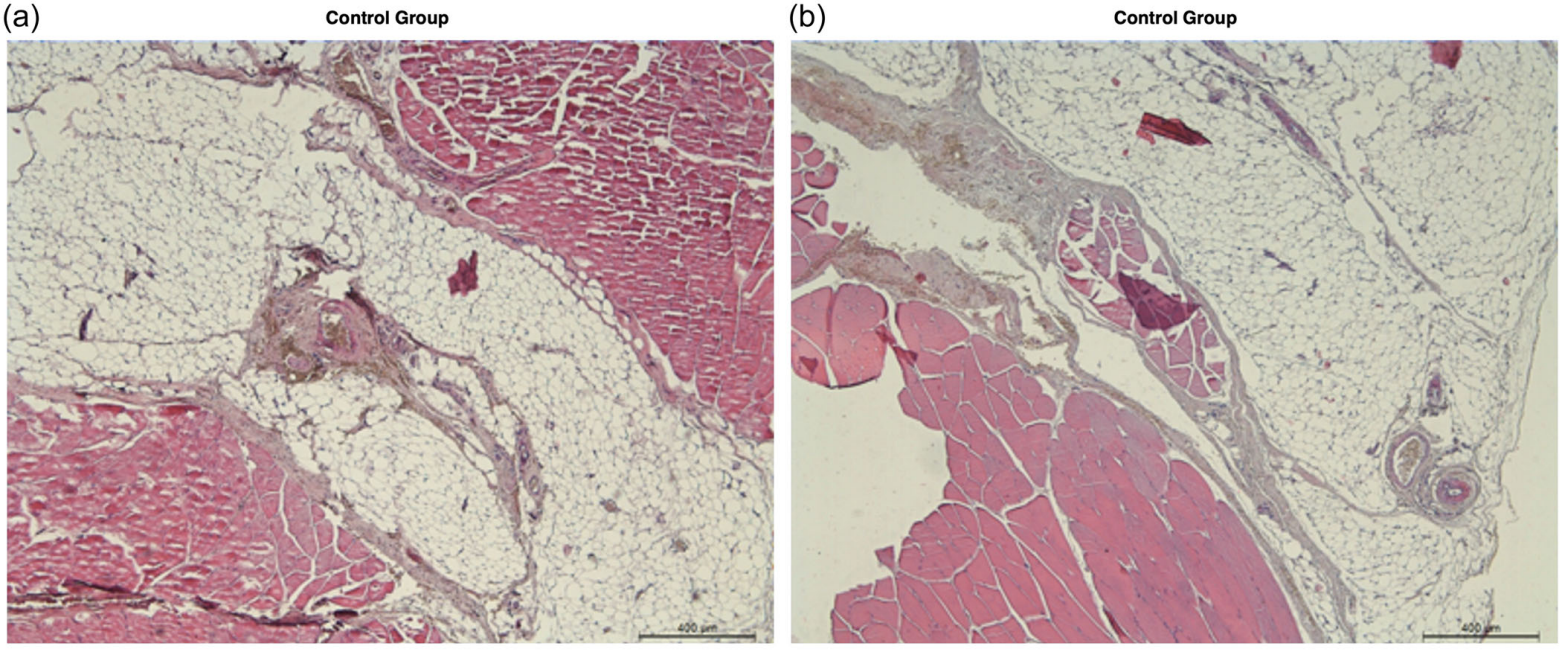
H&E stain ×5 magnification microscopic images of the control group supraspinatus muscles from the middle and distal muscle belly zones, respectively. (a) Control group: Large bodies of adipose tissue islands evident within the middle muscle belly zone. Small areas of muscle fibres within the adipose tissue are seen near the middle of the figure. (b) Control group: Advanced fatty degeneration with small muscle fibres and the distal muscle belly zone replaced mainly by adipose tissue, signifying the successful fatty degeneration model. H&E, haematoxylin–eosin.

## DISCUSSION

The most important finding of this study was that treatment of chronically fatty degenerated RC with surgical repair, and SM and MSC injection resulted in increased myogenesis, significantly decreased adipogenesis and improved tendon healing in the rat model. The intervention increased gross muscle mass and decreased fatty tissue with superior tendon healing. Furthermore, the histomorphometric analysis yielded improved values in the muscle tissue ratio and decreased adiposity ratio in the repair + SM and MSC group compared to the repair only group.

One of the main challenges in RC repair is the inability to obtain good tendon‐to‐bone healing and prevention of fatty degeneration with the current surgical techniques [10, 21, 26]. Stem cell‐based treatments might facilitate this healing process [10, 21, 26]. Previously, MSCs, perivascular stem cells (PSCs) and adipocyte‐derived stem cells (ADSCs) have all been proposed to improve RC repair outcomes and decrease the fatty degeneration of the affected muscle [6, 7, 14, 24, 27, 36]. Various reported methods exist to obtain stem cells, such as bone marrow aspiration, lipoaspiration or lower extremity muscle biopsies [6, 7, 14, 27]. These may be used in animal testing from various anatomic locations; however, performing these procedures at separate anatomic sites in humans may increase the overall complications, decrease patient satisfaction rates and be impractical to perform alongside arthroscopic tendon repair surgery. While attempting to improve the current treatment modalities, avoiding additional invasive procedures at sites other than the shoulder is desirable.

The treatment model in the current study was specifically designed to obtain SMs from the deltoid region, which is directly adjacent to the RC tendons and conventional shoulder arthroscopy portals. This method of cell harvest and treatment can be more easily adaptable to clinical setup and be more convenient in humans when compared to other established methods that require the dissection of separate anatomic zones such as the hind leg muscles, bone marrow aspirates from tibia or femur or inguinal fat pad [6, 7, 14, 27]. MSC isolation may require an additional biopsy of the abdominal region; however, abdominal adipose tissue is readily accessible in humans, and this procedure can be done prior to the arthroscopic repair surgery to enable cell cultivation and the injection of MSCs during the repair under direct arthroscopic visualization intraoperatively and under sonographic guidance postoperatively [4]. Furthermore, depending on the body habitus, the subcutaneous fat tissue can also be obtained from the surgical regions in humans during or prior to surgery [32]. This could not be performed in the current study because the deltoid region adipose tissue of the rats is much smaller in size compared to humans. Furthermore, the harvested tissue in the present study came from 2 rats and had to be used for cell culture and injection into 12 rats, necessitating more tissue.

Previously, Güleçyüz et al. reported that treatment of the rat supraspinatus tendon tear with repair + cultivated myocytes improved the fatty degeneration significantly compared to repair only or other stem cell therapies [14]. In their study, muscle fattening was provoked for 28 days by delaying the surgery, whereas, in our model, this duration was 8 weeks or 56 days, which created a chronic tear model with significant fatty degeneration. Similarly, Eliasberg et al. reported significantly decreased fatty degeneration with repair + PSC in a mouse model; however, in their study, there was no waiting period to provoke fatty degeneration of the RC before treatment [6]. The absence of a waiting period may have caused the tear model to behave more like an acute tear treated immediately, which may differ from our chronic fatty degeneration model.

In the current study, the tendon ratios of the repair only and repair + SM and MSC groups were comparable (*p* = 0.85), which may signify that repair is what facilitates the regeneration of the tendon tissue. However, the differences in the percentages of adipose tissue ratios and absolute muscle mass between the repair + SM and MSC and repair only groups significantly differed in favour of the repair + SM and MSC groups (*p* = 0.009 and *p* < 0.001). The myocyte contribution from the SMs and the generative capacity of the MSCs may have facilitated the decrease in adipogenesis and induced increased myocyte formation.

In one of the pioneering studies in stem cell application in the fatty degeneration model, Oh et al. showed that compared to repair only, repair + ADSC application improves tendon strength in the biomechanical measurements and decreases fatty infiltration in the histological analysis [27]. The current study has utilized histomorphometric analysis to evaluate the adipose, muscle and tendon tissue ratios in the supraspinatus samples. This method has been used previously in various types of tissues successfully and enables calculation of the areas of tendon, muscle and adipose tissue and measures the effect of the interventions on fatty degeneration quantitatively at the microscopic level [5, 22]. Additionally, this method helps evaluate the tendon tissue ratio along with the adipose tissue ratio, which may be significant as this portion is crucial for muscle function.

The histological examination of the supraspinatus tendons stained with H&E and TCM stains revealed several important findings, including less inflammation, decreased adipose tissue and more advanced healing with stem cell application. There was less inflammation than in the repair only group, as evidenced by decreased inflammatory cells in the repair + SM and MSC group. It was found in the current study that there was less adipose tissue in the group treated with stem cells, which is in contrast to results from Güleçyüz et al. [14] and Oh et al. [27]. While there was no control group with MSCs and without SMs in the current study, the difference might be due to the use of SMs which have been previously shown to be a valuable reservoir of muscle tissue in the treatment of myocardial tissue in animal models [3, 28]. In the current study, the control group showed no signs of healing and advanced fatty degeneration, signifying the necessity of surgical treatment for healing. The healing process appeared to be most advanced in the repair + SM and MSC group, with the highest amount of collagen fibres and tendon tissue and the lowest amount of adipose tissue. The repair + SM and MSC groups have shown improved collagen organization and superior structural integrity of the musculotendinous junction zone compared to repair only.

Previous studies have suggested that collagen fiber disorganization following tears in rat models peaks at around 2–4 weeks and may persist as long as a resin or polypropylene sutures were used to prevent reattachment, as was done in the current study [12, 16, 18]. The return of collagen structure to an organized state in the repair + SM and MSC group compared to the repair only group may indicate that stem cells can facilitate the connective tissue remodelling process. Treatment with repair + SM and MSCs has been previously proposed to affect fibrocartilage fibre orientation positively, and a similar effect may have been the reason that mature and organized collagen fibres were more prevalent in the repair + SM and MSC group in the current study [15]. Recently, inflammatory response after RC tear has been shown to peak around 5–7 days after injury and persist even after 28 days despite an early increase in satellite cells favoring regeneration [34]. In the current study, the histological analysis revealed decreased inflammatory cells in the repair + SM and MSC group, possibly due to additional regenerative and regulatory capacity provided by the injected stem cells and the local tissue response to repair.

One of the strengths of the current study was the well‐designed chronic degeneration model, which produced a successful chronic degeneration model evident in the control group which underwent an eight week waiting period and no further treatment. Another novelty was the SM isolation method from the deltoid region model, which may facilitate further research. Lastly, the study's detailed histological analysis of the supraspinatus muscles detailing the treatment response of the tissues at a cellular level was a strength.

This study had its limitations. Firstly, molecular methods for studying the viability and differentiation of the MSCs injected into the tissues were not included in the study's design; these methods could have been helpful to assess better the fate of the stem cells injected into the healing RC. In addition, a biomechanical analysis was absent, which could have provided load‐to‐failure values. However, this was not performed in favour of performing histomorphometric analysis in the intact supraspinatus muscles.

## CONCLUSION

In conclusion, we have shown that RC repair + SM and MSC injection produced a significant increase in muscle mass and a significant decrease in the adipose tissue ratio in the chronic RC fatty degeneration rat model. Surgical repair with adipose tissue‐derived MSC and deltoid region‐derived SM injections was significantly better than repair alone and could be a helpful treatment method.

## AUTHOR CONTRIBUTIONS

Material preparation, data collection and analysis were performed by Tahir Koray Yozgatli, Elif Gelenli Dolanbay, Tunca Cingoz, Ahmet Emre Paksoy and Baris Kocaoglu. The first draft of the manuscript was written by Tahir Koray Yozgatli, and all authors commented on previous versions of the manuscript. *Conceptualization*: Baris Kocaoglu, Unal Uslu and Ercument Ovali. *Methodology*: Baris Kocaoglu. *Formal analysis and investigation*: Tunca Cingoz, Ahmet Emre Paksoy, Elif Gelenli Dolanbay and Tahir Koray Yozgatli. *Writing—original draft preparation*: Tahir Koray Yozgatli, Elif Gelenli Dolanbay and Baris Kocaoglu. *Writing—review and editing*: All authors. *Funding acquisition*: Baris Kocaoglu, Ercument Ovali and Unal Uslu. *Resources*: Baris Kocaoglu, Ahmet Emre Paksoy and Unal Uslu. *Supervision*: Baris Kocaoglu. All authors contributed to the study's conception and design. All authors have read and approved the final manuscript.

## CONFLICT OF INTEREST STATEMENT

The authors declare no conflict of interest.

## ETHICS STATEMENT

This study was performed on an animal model; the ethical principles of experimenting on animal models were followed to replace, reduce and refine the experiment procedure to cause the least possible effect on animals. Thus, this study was approved by the Research Ethics Commission of Acibadem University for Animal Experiments, ACU‐HADYEK 2018/43.

## Supporting information

Supporting information.

## Data Availability

The data that support the findings of this study are available from the corresponding author (Baris Kocaoglu), upon request.
