# Supplementary material for: Satellite myoblast and mesenchymal stem cell injections decrease fatty degeneration after rotator cuff tear in rats
Source: J Exp Orthop. 2024 Jul 24;11(3):e12087. doi: 10.1002/jeo2.12087 (PMC11267176; doi:10.1002/jeo2.12087)
Supplement: Supplementary file 1 — Supporting information. [file JEO2-11-e12087-s001.pdf]

Supplemental File – Images and Descriptions of the Histological Samples

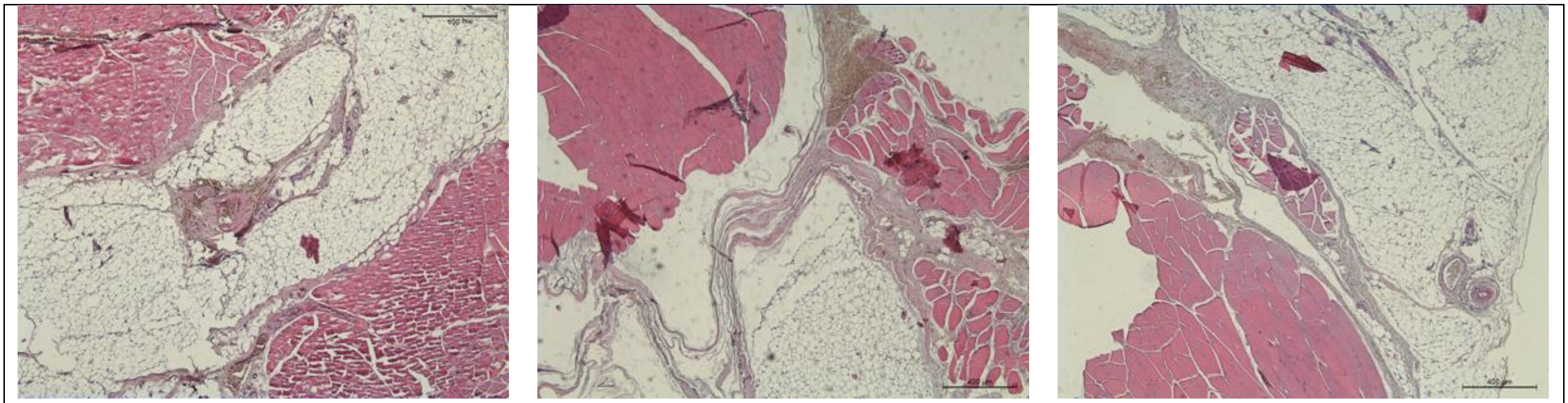

Image 1. A | Image 1. B | Image 1. C

Image 1: Control Group H&E Stain 5X magnification microscopic images of the supraspinatus muscles. Image 1. A: Degenerative muscle cells, increased adipose tissue within the muscle, narrow tendon tissue area with congestion. Image 1. B: Thin tendon region with congestion. Figure 1. C: Increased adipose tissue due to fatty degeneration, loose muscle tissue due to muscle atrophy.

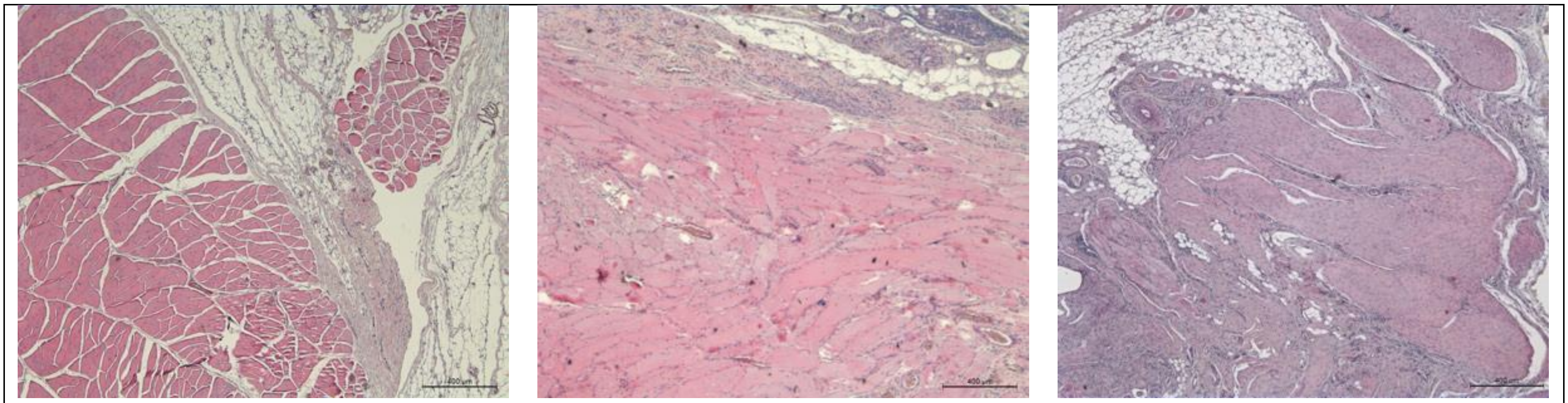

Image 2. A | Image 2. B | Image 2 .C

Image 2: Repair only Group: H&E Stain 5X magnification microscopic images of the supraspinatus muscles. Image 2 . A: Thickened tendon with increased collagen. Less adipose tissue compared to the control group. Image 2 . B & 2 . C: Mononuclear cell infiltration within the tendon and ongoing inflammatory processes are evident. Thickened tendon with a moderate increase in vascularization.

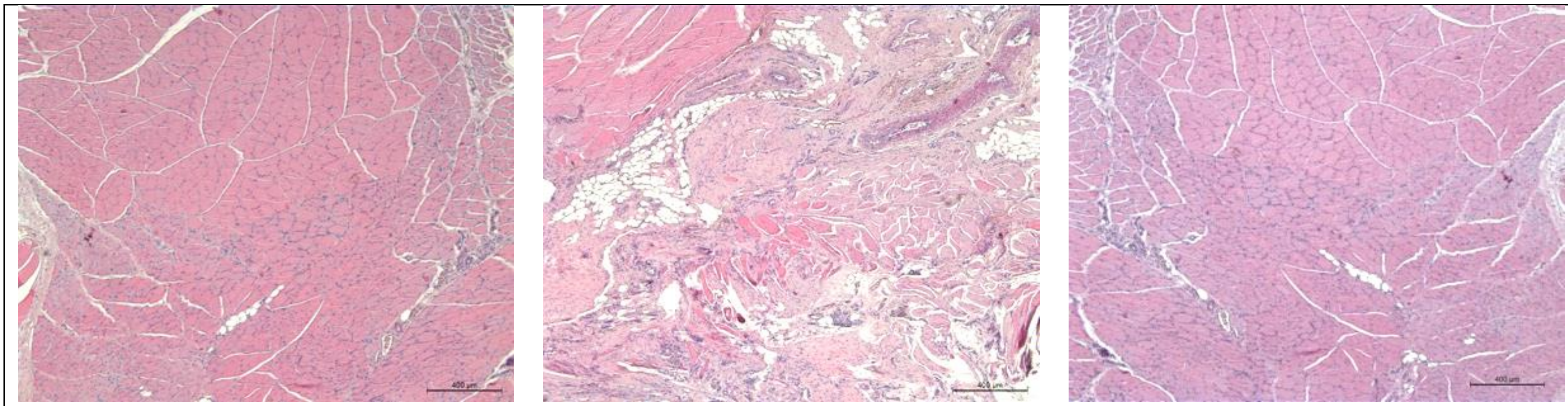

Image 3. A Image 3. B Image 3. C

Image 3: Repair + SM & MSC Group: H&E Stain 5X magnification microscopic images of the supraspinatus muscles. Image 3. A: Increased number of fibroblasts between the muscle fibers, markedly less adipose tissue within the muscle compared to the control and surgery only group, proper muscle tissue organization compared to the control and surgery only group. Image 3. B & 3. C: Tendon tissue and muscle tissue show mature integration with a marked increase in vascularization. Cellular hypertrophy in the connective tissue enveloping the muscle tissue.

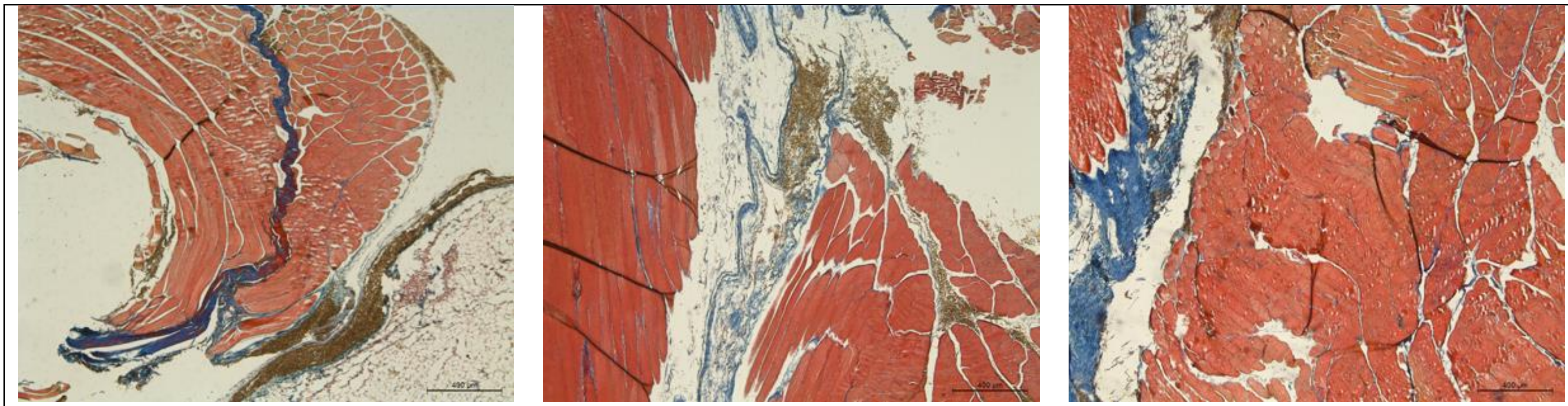

Image 4. A Image 4. B Image 4. C

Image 4: Control Group: TCM Stain 5X magnification microscopic images of the supraspinatus muscles. Image 4. A: Marked congestion, degenerative appearance in the muscle tissue, thin tendon with low collagen content. Image 4. B: Congestion between muscle fibers and in the perimysium. Image 4. C: Immature collagen fibers, congestion between the adipose tissue and tendon tissue intersection, and interstitial edema.

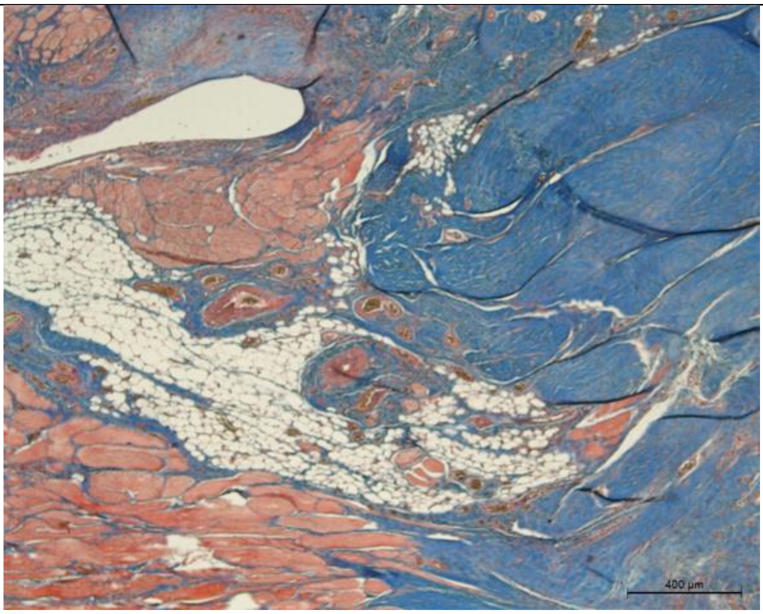

Image 5. A

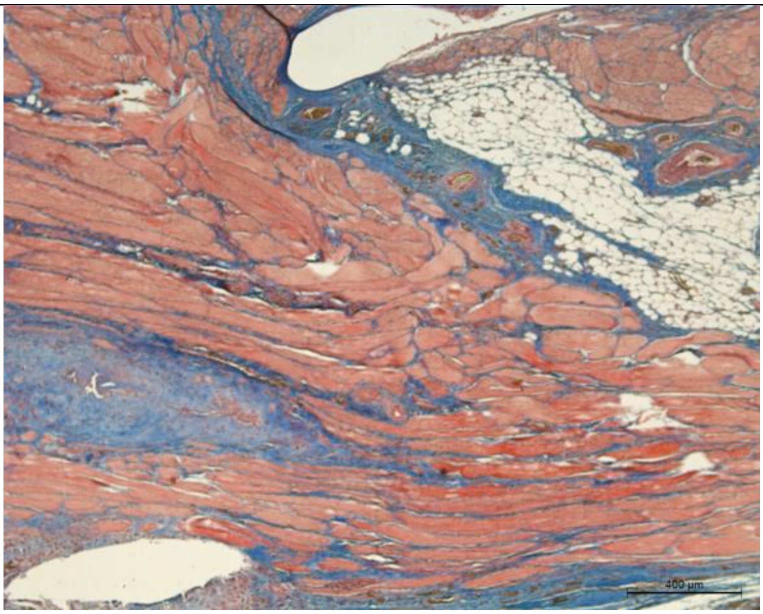

Image 5. B

Image 5: Repair only Group: TCM Stain 5X magnification microscopic images of the supraspinatus muscles. Image 5.A & 5 . B: Connective tissue integration has begun, collagen forming the tendon mostly immature, less adipose tissue compared to the control.

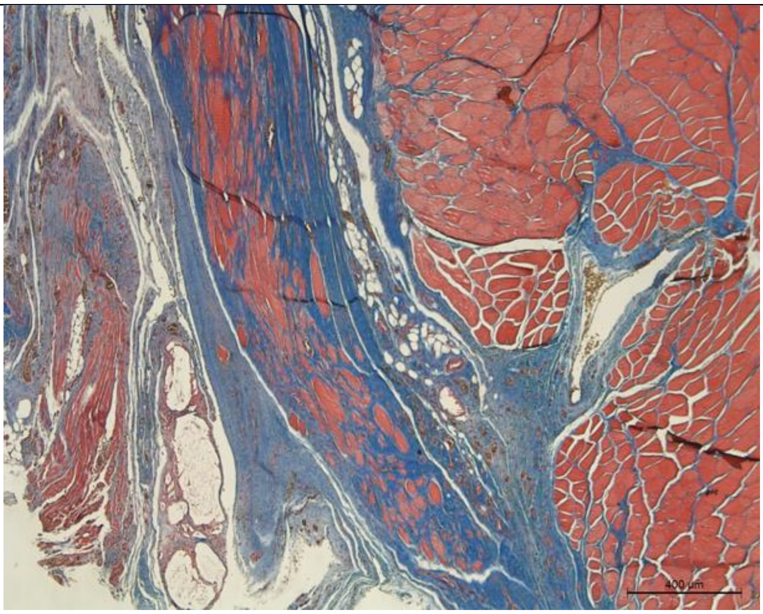

Image 6 .A

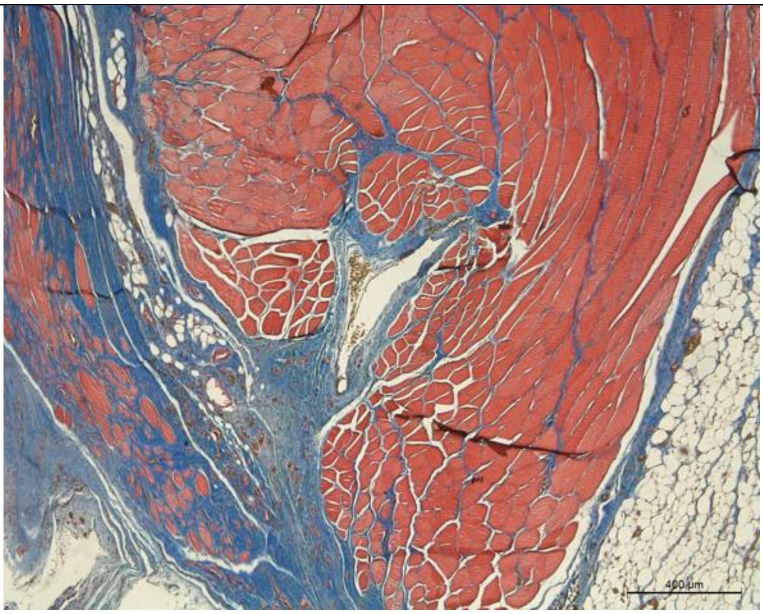

Image 6 .B

Image 6: Repair + SM & MSC Group: TCM Stain 5X magnification microscopic images of the supraspinatus muscles. Image 6.A & 6 . B: Well-developed connective tissue integration, muscle tissue and tendon tissue

containing mostly mature collagen fibers, markedly less adipose tissue compared to control and Surgery only groups.

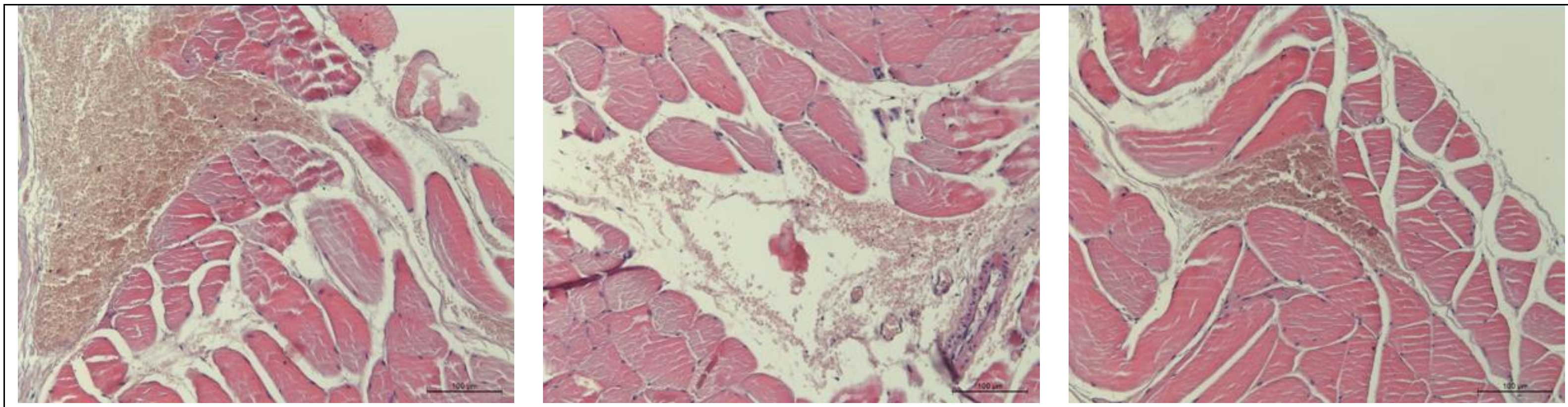

Image 7. A Image 7. B Image 7. C

Image 7: Control Group: H&E Stain 20X magnification microscopic images of the supraspinatus muscles. Image 7. A, 7. B & 7. C: Congestion between myocytes, necrotic changes in the muscle tissue, and apparent edema between myocytes.

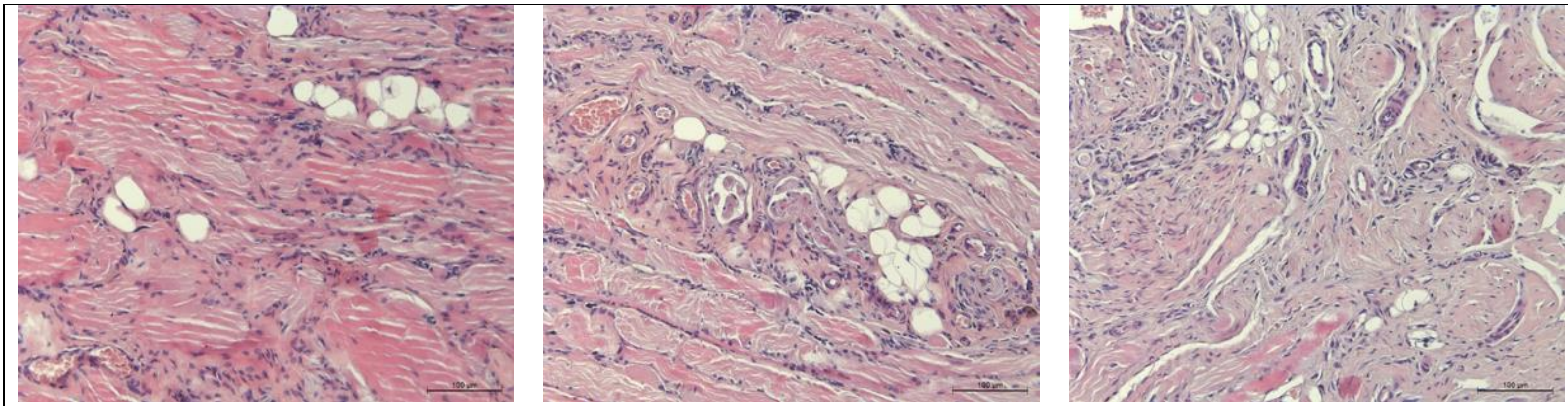

Image 8. A Image 8. B Image 8. C

Image 8: Repair Only Group: H&E Stain 20X magnification microscopic images of the supraspinatus muscles. Image 8. A: Adipocytes within the muscle tissue increased number of myocytes compared to the control group. Image 8. B & 8 . C: Connective tissue cells in groups, apparent vascularization, and increased angiogenesis compared to the control group.

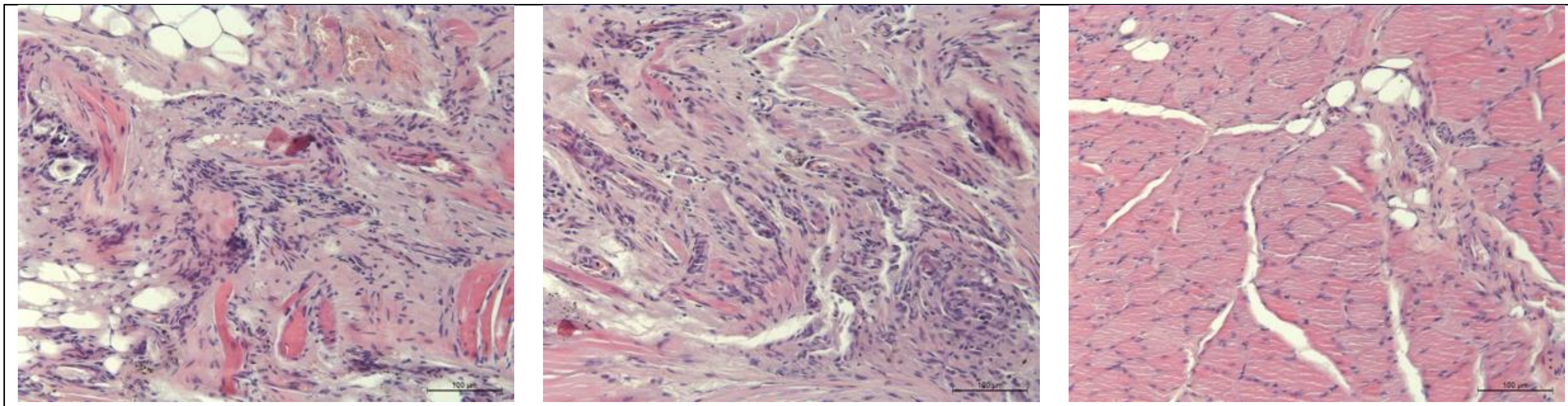

Image 9. A Image 9. B Image 9. C

Image 9: Repair + SM & MSC Group: H&E Stain 20X magnification microscopic images of the supraspinatus muscles. Image 9 . A, 9 . B & 9 . C: Markedly increased connective tissue cells, including fibroblasts and mature collagen bundles between the cells.

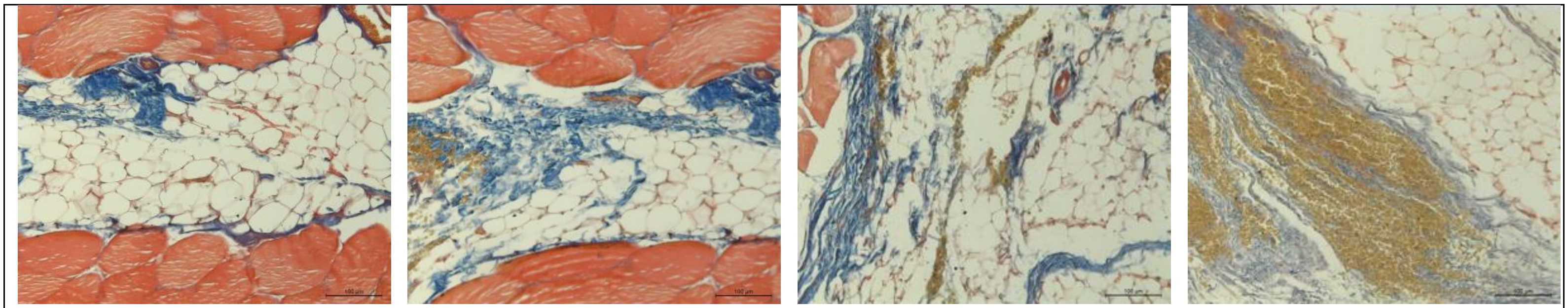

Image 10. A Image 10. B Image 10. C Image 10.D

Image 10: Control Group: TCM Stain 20X magnification microscopic images of the supraspinatus muscles. Image 10. A: Muscle tissue and adipose tissue in similar amounts, Tendon tissue is sparse, and the tendon is thin. Image 10.B: In addition to findings in Image 10. There is congestion. Image 10. C: Thin collagen fibers within the adipose tissue. Image 10.D: Marked congestion is observed.

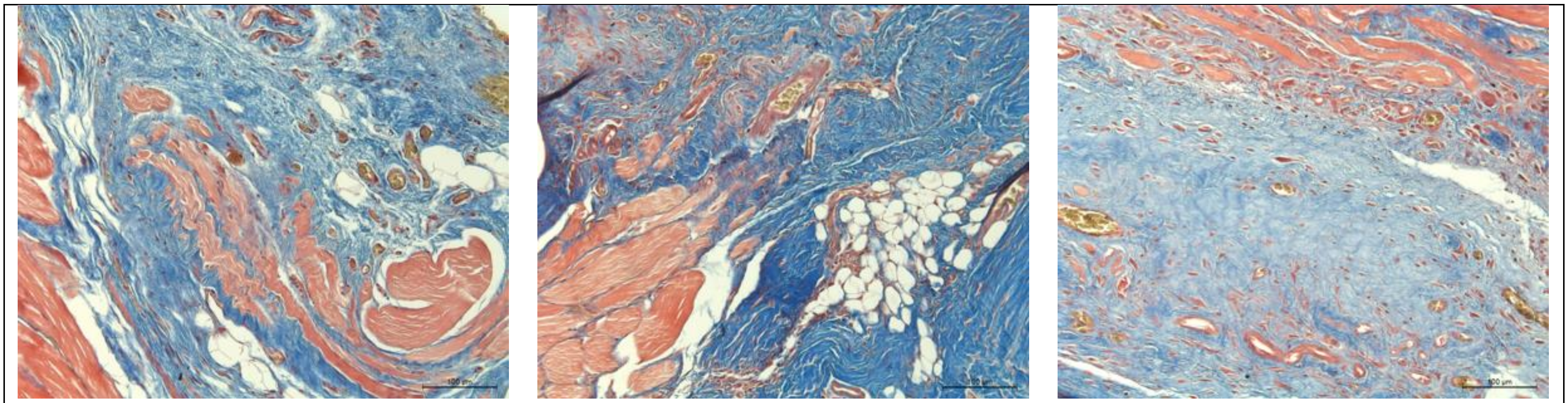

Image 11. A Image 11. B Image 11. C

Image 11: Repair Only Group: TCM Stain 20X magnification microscopic images of the supraspinatus muscles. Image 11.A & 11. B: Muscle and tendon intersection sites, showing increased vascularization in Image 11.A and apparent adipose tissue in Image 11.B. Image 11. C: Tendon tissue with mostly immature collagen fibers.

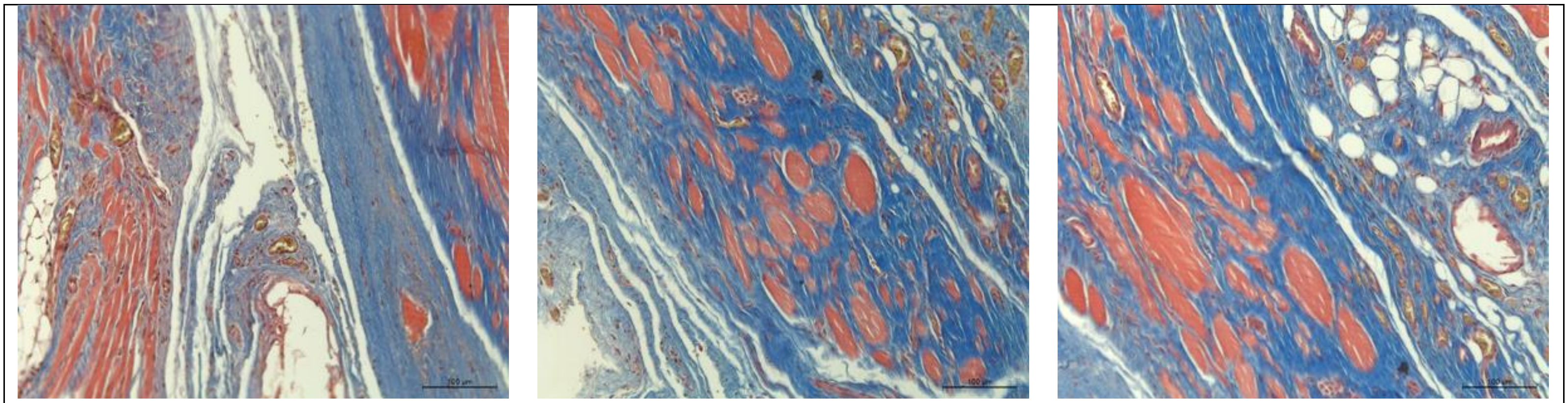

Image 12. A Image 12. B Image 12. C

Image 12: Repair + SM & MSC Group: TCM Stain 20X magnification microscopic images of the supraspinatus muscles. Image 12. A, 12. B & 12 . C: Advanced integration of the muscle tissue and tendon tissue. Mostly mature collagen bundles.

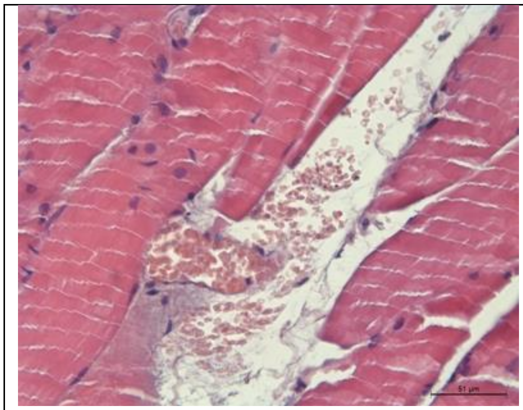

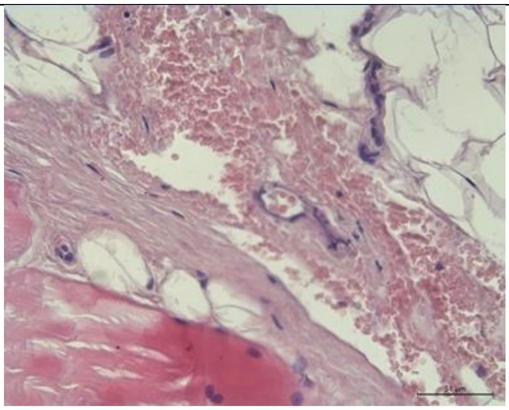

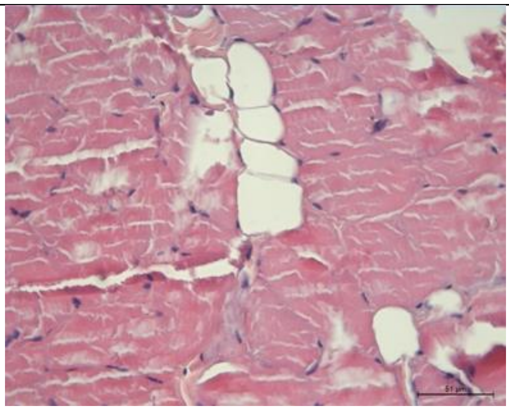

Image 13 .A Image 1 3.B Image 1 3.C

Image 13: Control Group: H&E Stain 40X magnification microscopic images of the supraspinatus muscles. Image 13 .A, 1 3.B & 1 3.C: Congestion and myocytes with degenerative and necrotic changes. Apparent edema between myocytes.

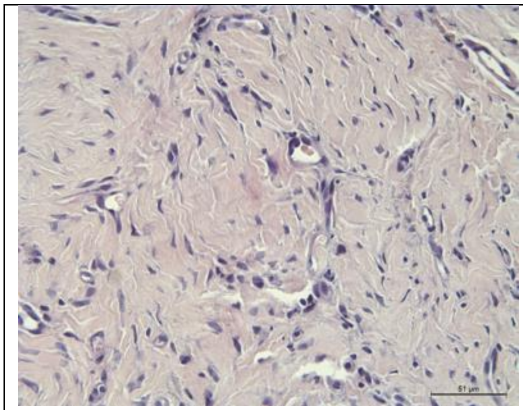

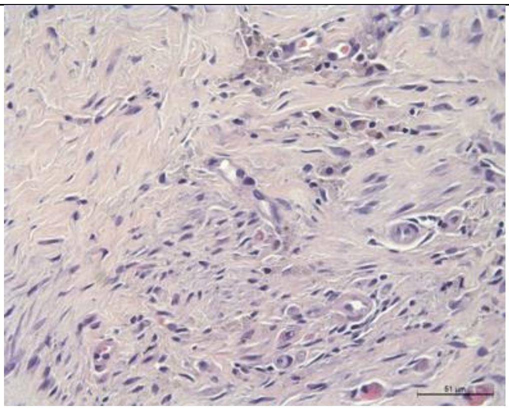

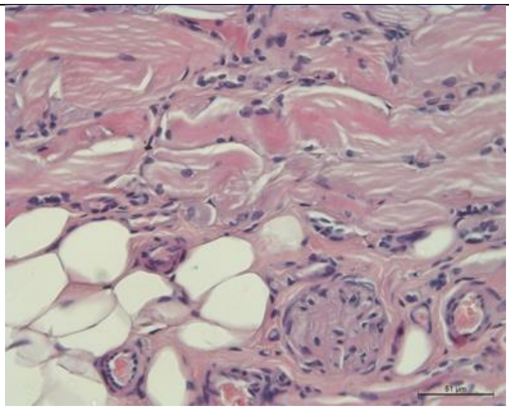

Image 14. A Image 14. B Image 14. C

Image 14: Repair Only Group: H&E Stain 40X magnification microscopic images of the supraspinatus muscles. Image 14. A, 14. B & 14. C: Fibroblasts and tenocytes are present along with mononuclear inflammatory cells. Adipocytes and myocytes are present.

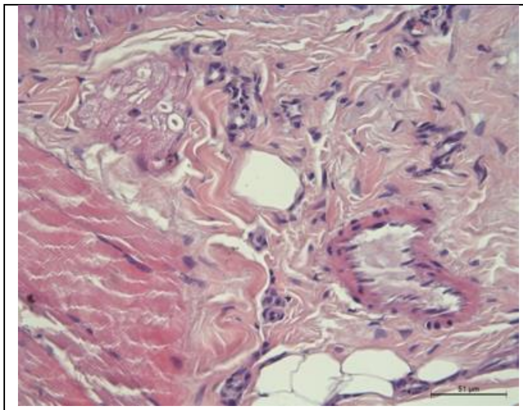

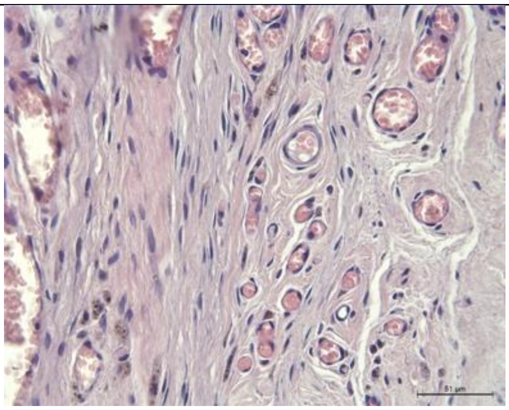

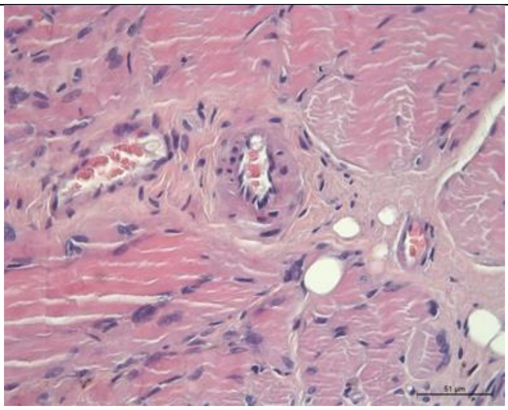

Image 15. A Image 15. B Image 15. C

Image 15: Repair + SM & MSC Group: H&E Stain 40X magnification microscopic images of the supraspinatus muscles. Image 15. A, 15. B & 15. C: Active fibroblasts and new small vessels are present.

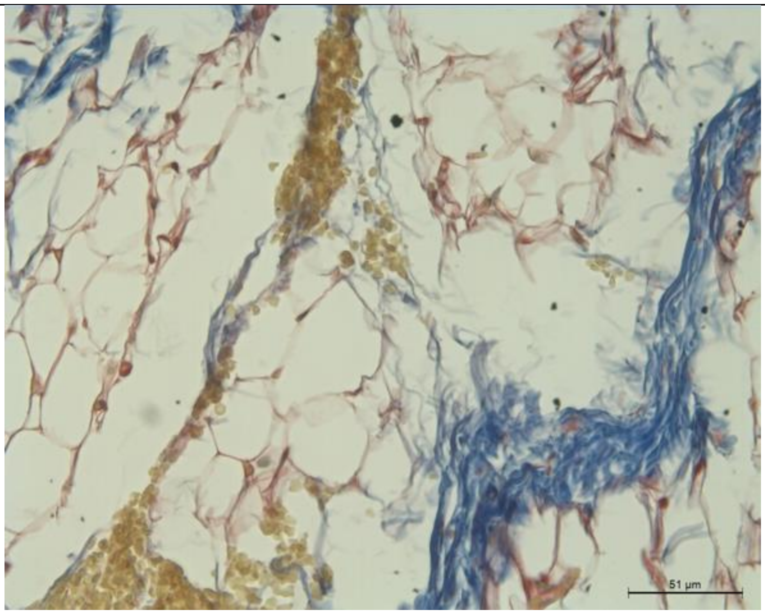

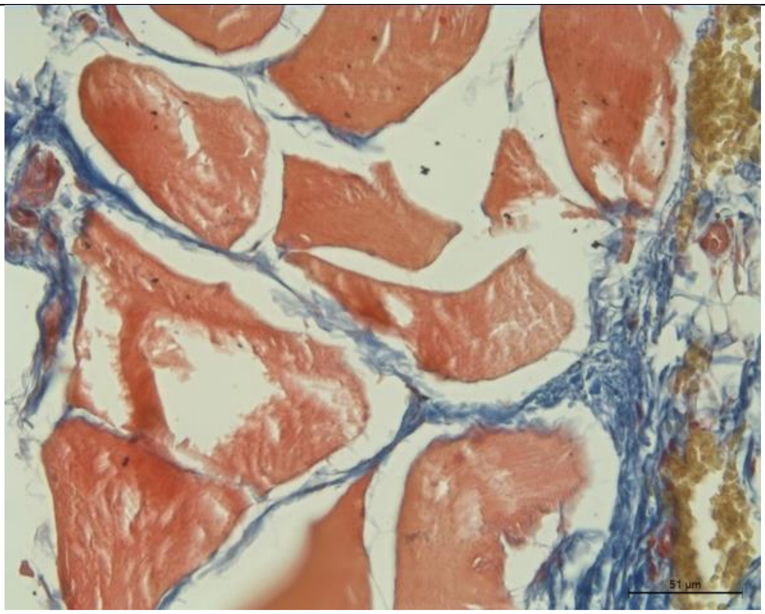

Image 16. A Image 16 .B

Image 16: Control Group: TCM Stain 40X magnification microscopic images of the supraspinatus muscles. Image 16. A: Congestion, thin collagen bundle, s, and increased adipose tissue. Image 16. B: Swollen myocytes.

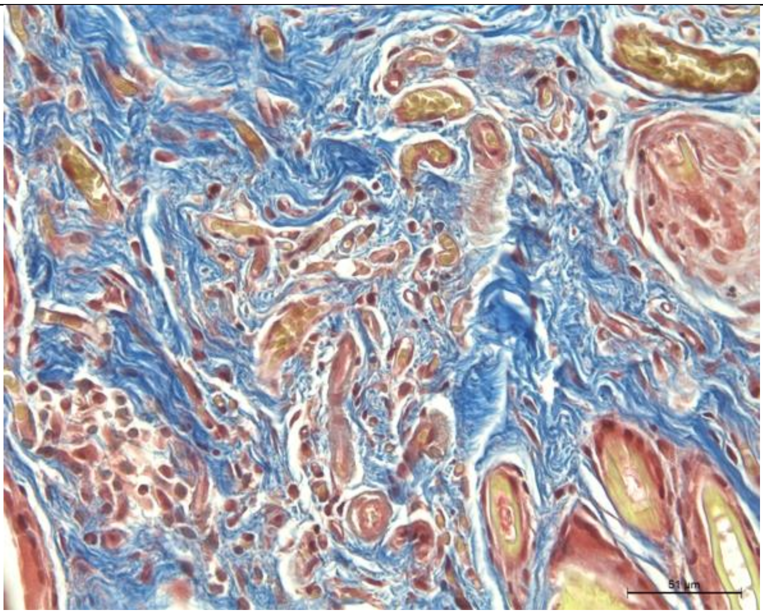

Image 17 .A

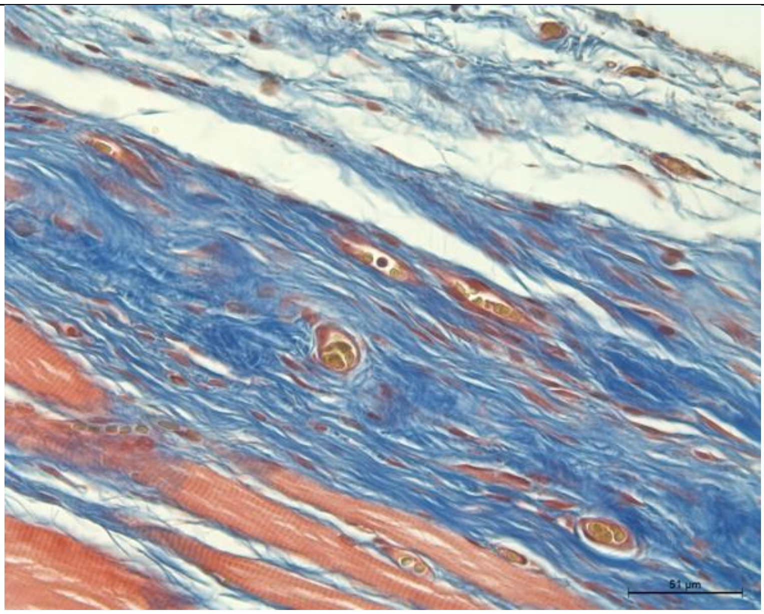

Image 17 .B

Image 17: Repair Only Group: TCM Stain 40X magnification microscopic images of the supraspinatus muscles. Image 17.A & 17. B: Fibroblasts and tenocytes along with inflammatory mononuclear cells are present. An apparent increase in vascularization and mature and immature collagen bundles are present. Fibrillary structure within myocytes (actin/myosin structure)

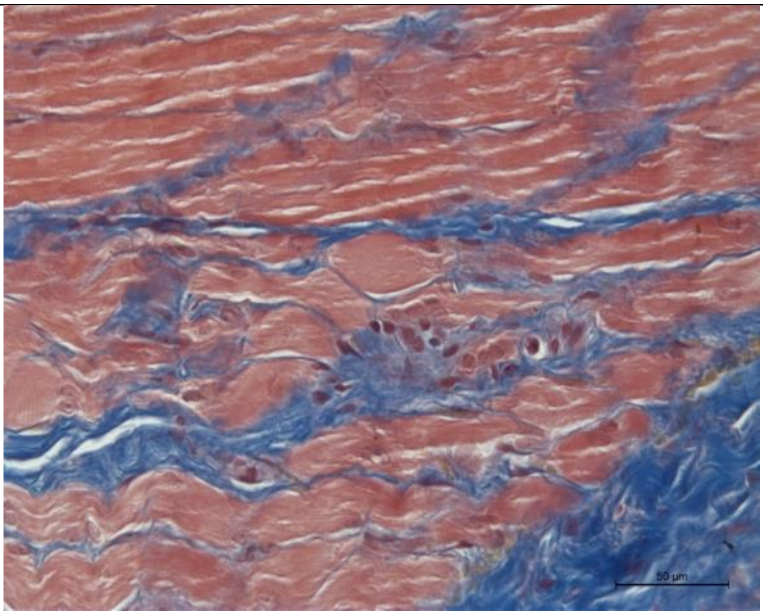

Image 18. A

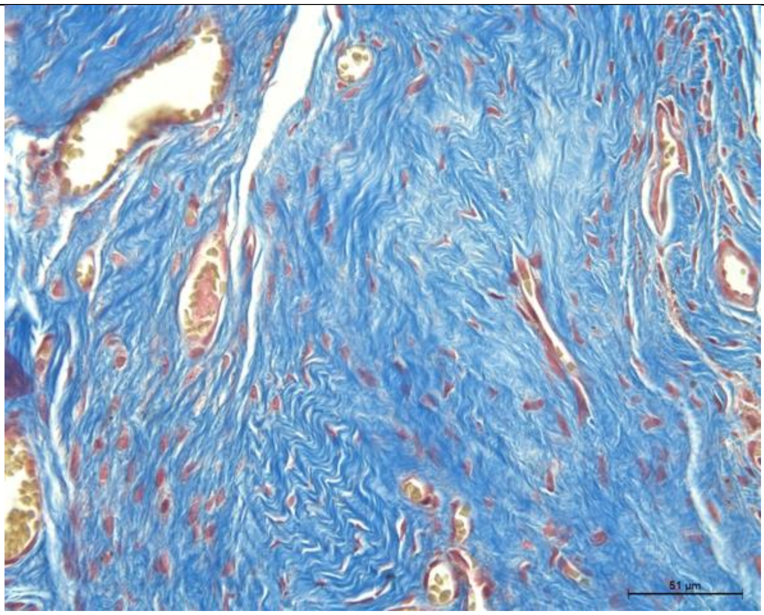

Image 18. B

Image 18: Repair + SM & MSC Group: TCM Stain 40X magnification microscopic images of the supraspinatus muscles. Image 18.A & 18. B: Active fibroblasts and tenocyte groups within mature collagen bundles.
